# Satisfaction with HyFlex Teaching and Law-abiding Leadership Education in Hong Kong University Students Under COVID-19

**DOI:** 10.1007/s11482-022-10040-4

**Published:** 2022-02-21

**Authors:** Daniel T. L. Shek, Xiaoqin Zhu, Xiang Li, Diya Dou

**Affiliations:** Department of Applied Social Sciences, The Hong Kong Polytechnic University, Hung Hom, Hong Kong

**Keywords:** HyFlex teaching, University students, Course evaluation, Leadership education, National security

## Abstract

Since the early days of COVID-19, university teaching has changed from face-to-face format to online mode. With the gradual containment of the pandemic, there is no need for school lockdown. As a result, the teaching format has changed to HyFlex mode integrating both face-to-face and online modes. Obviously, it is necessary to understand the academic quality of life among students under the Hyflex teaching mode. In this paper, we report an evaluation study on a leadership subject in Hong Kong delivered via HyFlex teaching using a post-lecture evaluation strategy. In one of the lectures, we covered law-abiding leadership in university students, including abiding by the Hong Kong National Security Law. The post-lecture evaluation showed that students generally held positive views toward the HyFlex teaching and they perceived that the subject promoted their well-being indexed by psychosocial competence. Regarding the lecture on law-abiding leadership, students agreed that the lecture promoted their psychosocial competence, personal development, knowledge about law-abiding behavior and national security (including the Hong Kong National Security Law), and readiness to serve as socially responsible leaders. Positive perceptions of the lecture design, teacher performance, lecture content of law-abiding leadership and national security, and benefits positively predicted students’ overall satisfaction with the lecture on law-abiding leadership and national security.

The preparation of this paper is financially supported by Wofoo Foundation and Li and Fung Endowed Professorship in Service Leadership Education. As I am the Editor-in-Chief of ARQOL, I have conflict of interest. Hence, I have invited Dr. Graciela Tonon (Book Reviewer Editor) to be the Action Editor who will invite two anonymous reviewers to review the paper.

## Introduction

COVID-19 has created unprecedented challenges for many people, including students. During the pandemic, cities and schools are locked down and students have to rely on online learning. Online learning is an alternative for continuing learning under COVID-19. Despite its flexibility and avoidance of physical contact, online learning has disadvantages. Primarily, students have to sit in front of the computer for long periods in online lectures under COVID-19, which may lead to physical health problems, such as a lack of exercise, dry eyes, and bodily pain. In addition, online teaching and learning make it difficult for students to form study groups, which may create social isolation. Furthermore, as pointed out by Shek (2021), online learning would be a concern to students who suffer from the digital divide as they may have to compete for resources like WIFI and computers with their family members, thus these students may be disadvantaged in online learning.

With the gradual containment of the pandemic and availability of vaccines, COVID-19 has stabilized in some parts of the world, which results in the resumption of face-to-face teaching. Nevertheless, because of travel restrictions and quarantine requirements, some international students may not be able to commence on-campus learning. Other students who are unsuitable to get vaccinated also have the problem of going back to the campus because most universities make vaccinations a precondition for students and staff to enter the campus. Furthermore, due to the unpredictability of the pandemic, there is a need for a “Plan B” in case another wave of outbreak takes place. Hence, online teaching and physical lessons are simultaneously provided during the pandemic. As a result, teachers face two batches of students when they teach—one batch of students join the physical class and another batch of students attend the lesson online. Pedagogically speaking, this kind of teaching has been termed as “hybrid learning” (i.e., a combination of face-to-face teaching and synchronous online teaching) or “HyFlex teaching” (i.e., hybrid and flexible teaching where students can choose to attend either one teaching mode according to their needs).

Previous studies have discussed the pros and cons of online teaching. For example, Dhawan (2020) pointed out that while online learning has strengths (e.g., time and location flexibility, wide range of audience and courses, and immediate feedback), it also has some weaknesses (e.g., technical issues such as connectivity, learners’ computer competence, lack of interaction, and distraction). In essence, there are many challenges of online teaching, such as digital illiteracy, digital divide, and quality assurance in the online examination. Unfortunately, the evaluation findings of online teaching and learning are not conclusive. Martin et al. (2020) conducted a review of related studies from 2009 to 2018 and concluded that only 6.14% of the publications focused on “the theme of evaluation and quality assurance” (p. 10). In another review of the effect of online courses on cognitive and affective outcomes of the students, Tallent-Runnels et al. (2006) concluded that the findings could not “withstand rigorous scientific scrutiny, because of the flaws in the research designs and execution methods” (p. 109).

Compared to the number of studies on the effects of online teaching, there are relatively fewer studies on HyFlex learning (i.e., face-to-face teaching plus online teaching at the same time). As commented by Abdelmalak and Parra (2016), “research regarding students’ perceptions of HyFlex is limited” (p. 20). HyFlex learning is flexible where students are allowed to choose to study onsite or online, thus having the greatest reach of students. However, HyFlex teaching is challenging for both teachers and students. For the teacher, hybrid teaching is very taxing because the teacher has to take care of two groups of students (one group in the physical classroom and another group online). For the students, there may be an increased distraction because two batches of students are involved. A handful of studies have evaluated the HyFlex model of teaching. Miller et al. (2013) showed that students generally welcomed this model and felt that the teaching mode promoted their understanding of the subject matter. In a social work education context, Malczyk (2019) showed that the students were optimistic about HyFlex teaching while they perceived both benefits and challenges of this teaching mode. Kakeshita (2021) also showed that around 85% of the students were generally satisfied with the HyFlex teaching. Despite the overall positive evaluation findings, there are two reasons why we should do more research to evaluate the impact of HyFlex teaching. First, learning quality is part of student well-being that would influence their overall quality of life. Obviously, learning poorly under HyFlex mode would impair the academic performance and well-being of the students, such as creating study stress and negative emotions. Second, evaluation findings can help teachers to reflect on their teaching and empower them, which eventually contributes to teachers’ quality of life.

In this paper, we report the findings of an evaluation study utilizing the post-lecture evaluation strategy to examine student perceptions of a leadership subject entitled “Tomorrow’s Leaders” based on HyFlex teaching mode in Semester 1 of the 2021-22 academic year at a university in Hong Kong. Since the implementation of the new 4-year undergraduate program in 2012, this 3-credit leadership subject has been offered to students (Shek et al., 2020). Adopting the positive youth development approach, this subject aims to promote the intrapersonal and interpersonal leadership attributes among the undergraduate students using experiential learning, including reflective, collaborative and active learning strategies. The topics covered include self-leadership, resilience, emotional competence, moral competence, spirituality, social competence, team building, conflict resolution and promotion of well-being. Using different evaluation mechanisms, the findings indicated that students taking this subject showed improvement in psychosocial competencies, leadership attributes, and life satisfaction (Li & Shek, 2020; Yu et al., 2021). It also showed that students were generally satisfied with the subject and agreed that the subject was able to promote their holistic development in multiple areas, such as problem-solving skills and character development. Because of its innovative approach and positive evaluation findings, we were awarded the Silver Award (“Ethical Leadership”) under the QS Reimagine Education Awards 2017. We also won the University Grants Committee Teaching Award in 2018 in Hong Kong. Recently, we received the Gold Award in the Nurturing Well-being & Purpose category at QS Reimagine Education 2021.

Under COVID-19, we moved to online teaching in the 2019-20 academic year (second semester) and the 2020-21 academic year (first and second semesters). To understand the quality of teaching, we conducted post-lecture evaluations to understand students’ responses to online teaching and learning. Evaluation findings showed that students had a positive evaluation of the online mode of the subject, although they still wished to have face-to-face teaching (Shek et al., 2021, 2022). In Semester 1 of the 2021-22 academic year, the subject was delivered through HyFlex teaching and the routine post-lecture evaluations were carried out for all lectures, including a newly incorporated lecture on “law-abiding leadership.”

## Law-abiding Leadership Education and National Security Education

Because of the social event taking place in 2019-2020, there is a voice in the Hong Kong society questioning whether students have a strong awareness of law-abidingness. Hence, there is a call for universities to enhance students’ awareness of law-abidingness in university education in Hong Kong. As such, we developed a new 3-hour lecture on law-abiding leadership in the leadership subject as Lecture 8 plus seven hours of self-study on materials assigned by the teachers. Based on the argument that a leader should understand the law (Hong Kong National Security Law, HKNSL) as well as its context (including the concept of national security and historical development of modern China), the topics covered in the law-abiding leadership lecture include the “why there is a need of” and “what is” law-abiding leadership, the concept of national security, and the historical and political contexts of national security in Hong Kong (including modern Chinese history, Century of humiliation, the Constitution of People’s Republic of China, the Basic Law), and the HKNSL.

In Lecture 8, our approach is to introduce objective facts and rationales of the HKNSL to students and help them to understand the importance of the law from different perspectives. We encouraged and invited students to think about some public opinions, such as “some people in Hong Kong say that it is ‘unreasonable’ to impose the Hong Kong National Security Law in Hong Kong by the Central Government,” and “the Chinese political system is not ‘democratic’ in the Western sense, Chinese people do not have confidence about the laws enacted in China.” We also introduced the recent study conducted by the Ash Center for Democratic Governance and Innovation of Harvard Kennedy School which reported that a very high proportion of Chinese people credited the Central Government (Cunningham et al., 2020), and then asked students to reflect on the findings. In short, we take the stand that whether one obeys the law is a personal choice and we respect the choice made by every individual. However, as educators, we encourage, urge and admonish students to obey the HKNSL and they should clearly understand the consequences of committing offenses under the HKNSL.

As some topic in the law-abiding leadership lecture is highly politically sensitive, particularly the HKNSL, we have to understand the views of the students, including whether they endorsed the importance of law-abiding leadership and the coverage on national security as well as the HKNSL in the lecture. More importantly, it is essential to collect students’ opinions and to understand whether they perceived that the lecture contributes to their well-being, such as improvement in psychosocial competence. To this end, we used the post-lecture evaluation strategy to collect subjective outcome evaluation data. Teachers in the higher education sector have commonly used this strategy to understand the impact of subjects.

National security has emerged as a hot topic since the September 11 attacks in the United States. As terrorist attacks adversely affect the well-being of people in a society, it is important to re-visit the notion of national security to make a place more “secure”. Conceptually speaking, national security is a multi-dimensional concept covering many areas (such as economic security and food security), although it is commonly conceived in terms of military security. As a citizen, one should contribute to national security by obeying national security laws (such as anti-terrorism laws) and developing a sense of obeying the law. To achieve this goal, national security education (or homeland security education) has been regarded as a vehicle to promote the knowledge, attitude, and behavior of students regarding national security. In some countries, national security education is embedded in the high school curriculum in different forms, mostly in citizenship and civic education. For example, Singapore covers national security under national education, such as understanding the vulnerabilities and constraints of the country (Chia, 2015). In Britain, policy-makers have used the strategy of promoting “British values” to reduce radicalization in students (Winter et al., 2021). In the United States, there are even high schools implementing Homeland Security programs to provide vocational training for socially deprived students looking for jobs in national security (Nguyen, 2017).

Regarding national security education in the university context, there are three observations. First, there are discipline-specific subjects on national security, such as information technology (Bogolea & Wijekumar, 2004) and law (Silliman, 2005). Second, undergraduate programs are specifically designed to train students to be homeland security and emergency management professionals (Ramsay & Renda-Tanali, 2018; Stewart & Vocino, 2013). Third, there are few general education subjects on national security education, although liberal arts education has been considered a good way to safeguard national security, such as the preservation of the democratic political system. For example, McCarthy (2003) suggested that topics on homeland security should be promoted via distance learning. Spaulding and Parker (2019) also argued that civic education is the best answer to promote national security, although this is not well done. Bara et al. (2014) also pointed out that university teachers were pessimistic about ethics and civic education in European higher education and “the majority of the lecturers stated that they did not assess it, perhaps owing to a lack of training, the difficulty in locating evidence or its negligible academic weight” (p. 29).

In Hong Kong, because of social unrest in the 2019-2020 and failure to enact Article 23, the “Standing Committee of the National People’s Congress of the People’s Republic of China” passed the “Law of the People’s Republic of China on Safeguarding National Security in the Hong Kong Special Administrative Region” (also known as the “Hong Kong National Security Law,” HKNSL) on 30 June 2020. In Article 10 of the HKNSL, it is stated: “the Hong Kong Special Administrative Region shall promote national security education in schools and universities and through social organizations, the media, the internet and other means to raise the awareness of Hong Kong residents of national security and of the obligation to abide by the law.” Hence, universities in Hong Kong are obligated to introduce the HKNSL in Hong Kong. As the matter is politically sensitive, students may have negative reactions to national security education, such as fear, rejection, confusion, and ambivalence. Besides, there is a rumor of “brain-washing” students through national security education. To some extent, students’ impressions and reactions to the national security education constitute an important aspect of their quality of life on and outside the campus. As it is important to examine the quality of life in the context of education (Tonon, 2020a, b), we attempted to understand the views of students on national security education, which would shape their well-being. In this study, we asked several research questions as follows:

1. What are students’ perceptions of HyFlex teaching in the leadership subject? With reference to the previous studies (Li & Shek, 2020; Yu et al., 2021), it was expected that students would have positive perceptions of the lectures (Hypothesis 1). We tested this hypothesis in all lectures except for the lecture on law-abiding leadership (Lecture 8).
2. Regarding the 3-hour HyFlex lecture on law-abiding leadership (Lecture 8), what are students’ perceptions of the lecture content, teachers’ performance, teaching mode, and lecture benefits? It was expected that most of the students would give positive quantitative and qualitative responses (Hypothesis 2).
3. What factors would influence the overall perception of the lecture on law-abiding leadership (Lecture 8)? Based on the existing findings (Shek & Law, 2014), we expected that the perceived quality of the lecture (e.g., design and class attributes), teacher attributes, law-abiding leadership content, and benefits of the lecture would be positively related to the overall perception of the lecture (Hypotheses 3a to 3d).

## Method

### Participants and Procedures

In the first semester of the 2021-21 academic year, 1,146 students took the “Tomorrow’s Leaders” subject. As HyFlex teaching is a new teaching mode, we collected post-lecture evaluation to understand students’ experiences and comments on the lectures, including their views on the usefulness of HyFlex teaching. After each lecture (Lecture 1 to Lecture 11), students were invited to respond to an online post-lecture evaluation questionnaire anonymously and voluntarily. We did not collect post-lecture evaluation data for Lecture 12 and Lecture 13 because the two lectures were used for student presentations and discussion.

### Instruments

For all lectures except for Lecture 8, students responded to a 28-item post-lecture evaluation questionnaire which showed high internal consistency in different lectures (Table 1). The 28 items are as follows:

**Table 1:** Number of online post-lecture evaluation questionnaires used in reliability analyses and alpha values in different lectures

Lecture	Valid number	Cronbach’s Alpha	N of Items
Lecture 1	916	0.967	28
Lecture 2	790	0.977	28
Lecture 3	863	0.984	28
Lecture 4	810	0.985	28
Lecture 5	806	0.986	28
Lecture 6	851	0.987	28
Lecture 7	772	0.990	28
Lecture 8	813	0.991	27
Lecture 9	791	0.990	28
Lecture 10	787	0.991	28
Lecture 11	725	0.989	28

Note: Lectures 1-7 and Lectures 9-11 used the 28-item scale and Lecture 8 used a 27-item scale

1. “The design of this lecture was very good.”
2. “The online classroom atmosphere of this lecture was very pleasant.”
3. “This lecture increased my awareness of the importance of self-development.”
4. “This lecture has improved my problem-solving ability.”
5. “This lecture has improved my understanding of the importance of attributes of successful leaders (e.g., critical thinking, moral competence, etc.).”
6. “This lecture has improved my interpersonal communication skills.”
7. “There was much peer interaction amongst the students in this lecture.”
8. “This lecture has improved my critical thinking.”
9. “There was much student participation in this lecture.”
10. “There were many opportunities for reflection in this lecture.”
11. “This lecture is helpful to my personal development.”
12. “Overall speaking, I have a very positive evaluation of this lecture.”
13. “The lecturer had a good mastery of the lecture material.”
14. “The lecturer used different methods to encourage students to learn.”
15. “The lecturer in this lecture was able to help students understand the knowledge covered in the lecture.”
16. “The lecturer was able to effectively take care of the students who attend class in-person and students who join the class virtually.”
17. “Overall speaking, I have a very positive evaluation of the lecturer in this lecture.”
18. “The hybrid teaching of this lecture was very smooth.”
19. “The hybrid teaching enabled me to learn more efficiently about this lecture.”
20. “Compared to online teaching, the hybrid teaching approach provides me with more interaction opportunities with teachers and classmates.”
21. “Compared to the traditional teaching methods, the hybrid teaching approach provides me with more interaction opportunities with teachers and classmates.”
22. “Compared to the traditional teaching methods, the hybrid teaching approach increases my flexibility of access to the lecture.”
23. “Compared to traditional classroom learning, I learn better in hybrid learning-based environments.”
24. “Hybrid teaching allows me to learn the lecture materials according to my situation.”
25. “I prefer hybrid learning to traditional learning.”
26. “I prefer hybrid learning to online learning.”
27. “Overall speaking, I like the hybrid learning experience.”
28. “Overall speaking, I like the arrangement of this lecture (i.e., hybrid teaching and learning).”

To understand students’ views toward the lecture on law-abiding leadership, we modified the above original scale to form a 27-item measure with good reliability (see Table 1) as follows:

1. “The design of this lecture was very good.”
2. “The classroom atmosphere of this lecture was very pleasant.”
3. “This lecture increased my awareness of the importance of self-development.”
4. “This lecture has improved my problem-solving ability.”
5. “This lecture has improved my understanding of the importance of attributes of successful leaders (e.g., critical thinking, moral competence, etc.).”
6. “This lecture has improved my interpersonal communication skills.”
7. “There was much peer interaction amongst the students in this lecture.”
8. “This lecture has improved my critical thinking.”
9. “There was much student participation in this lecture.”
10. “There were many opportunities for reflection in this lecture.”
11. “This lecture is helpful to my personal development.”
12. “This lecture helps me understand the concepts of national security.”
13. “This lecture helps me understand the offenses and penalties surrounding the National Security Law in Hong Kong.”
14. “This lecture helps me understand the importance of law abidance in leadership.”
15. “Law abidance is important for the stability of a society.”
16. “I will try my best to serve as a socially responsible leader.”
17. “Overall speaking, I have a very positive evaluation of this lecture.”
18. “The lecturer had a good mastery of the lecture material.”
19. “The lecturer used different methods to encourage students to learn.”
20. “The lecturer in this lecture was able to help students understand the knowledge covered in the lecture.”
21. “The lecturer was able to effectively take care of the students who attend class in-person and students who join the class virtually.”
22. “Overall speaking, I have a very positive evaluation of the lecturer in this lecture.”
23. “The hybrid teaching of this lecture was very smooth.”
24. “The hybrid teaching enabled me to learn more efficiently about this lecture.”
25. “Hybrid teaching allows me to learn the lecture materials according to my situation.”
26. “Overall speaking, I like the hybrid learning experience.”
27. “Overall speaking, I like the arrangement of this lecture.”

Roughly speaking, there are several groups of items on this scale. These include lecture design and its implementation (“course”: items 1, 2, 7, 9, and 10), teacher attributes (“teacher”: items 18, 19, 20, 21, and 22), law-abiding leadership, and national security (“law abidance” items 12, 13, 14, 15 and 16), benefits (“benefits” items 3, 4, 5, 6, 8 and 11), and overall evaluation (“overall”: items 17, 26 and 27).

In addition to the 27 items, students were also invited to give their comments concerning the following instruction: “Please write down your comments on this lecture (e.g., what you have learned in this lecture). If there is no comment, please write NIL”. A comment was coded into different responses if appropriate (e.g., “the lecture was enjoyable and useful” were coded as “enjoyable” and “useful”). A response was coded as “positive,” “negative,” “neutral” (i.e., with both positive and negative elements), or undecided (i.e., cannot decide its positivity or negativity) according to its nature. The inter-rater reliability was excellent (Fleiss’ Kappa = 0.81, Landis & Koch, 1977).

## Results

Reliability analyses showed that the post-lecture evaluation scale was internally consistent across all lectures (Table 1). Regarding the lectures other than Lecture 8, frequency analyses showed that the evaluation findings are generally positive (Table 2). For the quality of the teachers (item 13 to item 17), the students had positive perceptions of the performance of the teachers. For instance, over 90% of the respondents agreed that the teachers encouraged and cared about the students, and they were well-prepared for the lectures. Students also held positive views on the lectures (items 1, 2, 7, 9, and 10), including the good design of the lectures, the good atmosphere of the classes, and the opportunities for interaction and reflection. Moreover, the students also perceived that the lectures were beneficial to them (items 3, 4, 5, 6, 8, and 11), including improvements in problem-solving, critical thinking, personal development, and understanding of the qualities of successful leaders. Finally, students were positive about the HyFlex teaching mode (items 18 to 28) and they preferred HyFlex learning to traditional face-to-face teaching and online teaching. In short, the findings provide support for Hypothesis 1 and indicate that students had positive perceptions of the different aspects of the subject.

**Table 2:** Means and positive responses rates of post-lecture evaluation for Lectures 1-7 and Lectures 9-11

Lecture		Lecture 1 (N=1043)		Lecture 2 (N=909)		Lecture 3 (N=979)		Lecture 4 (N=895)			Lecture 5 (N=893)	Lecture 6 (N=920)		Lecture 7 (N=842)		Lecture 9 (N=856)		Lecture 10 (N=846)		Lecture 11 (N=780)	
Questionnaire Item		*Mean*	Positive response rate%	Mean	Positive response rate%	Mean	Positive response rate%	Mean	Positive response rate%	Mean	Positive response rate%	Mean	Positive response rate%	Mean	Positive response rate%	Mean	Positive response rate%	Mean	Positive response rate%	Mean	Positive response rate%
Q1	The design of this lecture was very good.	4.84	95.55	4.79	95.11	4.88	96.37	4.96	96.61	4.93	96.6	4.99	97.14	4.99	95.68	5	95.77	5.09	97.51	5.1	97.17
Q2	The online classroom atmosphere of this lecture was very pleasant.	4.9	94.04	4.84	94.6	4.92	95.34	5.01	96.52	4.95	95.39	5.04	97.37	5	95.45	5.02	96.36	5.09	96.92	5.14	97.3
Q3	This lecture increased my awareness of the importance of self-development.	4.8	94.98	4.91	96.9	4.91	96.9	4.99	97.76	4.98	96.85	5.01	97.17	5.01	96.05	5.03	96.37	5.1	97.27	5.13	97.43
Q4	This lecture has improved my problem-solving ability.	4.55	91.14	4.72	93.15	4.84	95.24	4.93	96.75	4.93	95.5	4.96	96.51	4.94	95.21	4.99	95.89	5.08	96.67	5.11	96.39
Q5	This lecture has improved my understanding of the importance of attributes of successful leaders (e.g., critical thinking, moral competence, etc.).	4.88	96.05	4.92	96.78	4.91	96.81	4.97	97.19	4.91	95.96	5.05	97.6	5.02	96.05	5.05	96.61	5.11	97.27	5.15	97.43
Q6	This lecture has improved my interpersonal communication skills.	4.72	94.03	4.67	90.38	4.87	96.28	4.98	96.63	4.86	94.61	4.98	96.41	4.95	94.87	5.02	96.35	5.11	96.56	5.13	96.52
Q7	There was much peer interaction amongst the students in this lecture.	4.63	88.59	4.6	88.24	4.84	93.69	4.98	95.72	4.83	93.06	5.01	96.95	4.9	92.37	5	95.29	5.03	95.14	5.09	96.4
Q8	This lecture has improved my critical thinking.	4.52	88.68	4.7	93.1	4.81	95.34	4.91	95.94	4.9	95.38	5.01	96.84	4.96	95.12	5	96.13	5.05	96.91	5.11	97.18
Q9	There was much student participation in this lecture.	4.69	90.43	4.76	92.25	4.88	94.94	5.01	96.74	4.88	94.36	5.02	97.6	4.97	93.92	5.02	96.14	5.06	96.44	5.12	97.43
Q10	There were many opportunities for reflection in this lecture.	4.64	91.53	4.85	94.25	4.87	95.56	4.96	97.42	4.95	96.16	5.02	97.28	5.02	95.6	5.01	96.02	5.08	96.8	5.13	97.42
Q11	This lecture is helpful to my personal development.	4.74	93.5	4.91	95.66	4.9	96.8	5	97.18	4.98	96.7	5.02	97.49	5.03	95.47	5.03	96.59	5.1	97.03	5.15	97.18
Q12	Overall speaking, I have very positive evaluation of this lecture.	4.92	94.87	4.93	95.79	4.95	95.79	5.02	97.53	4.99	96.51	5.05	97.39	5.06	95.94	5.07	96.93	5.14	97.51	5.17	97.3
Q13	The lecturer had a good mastery of the lecture material.	5.08	97.97	5.05	97.33	5.02	97.11	5.03	97.19	5.07	98.09	5.08	98.04	5.09	96.3	5.09	97.43	5.14	97.74	5.18	98.07
Q14	The lecturer used different methods to encourage students to learn.	5	97.39	4.98	96.45	4.97	96.79	5.04	97.53	5.04	96.97	5.07	98.03	5.06	96.77	5.08	96.95	5.14	98.1	5.15	97.94
Q15	The lecturer in this lecture was able to help students understand the knowledge covered in the lecture.	4.96	97.87	5	97.33	4.98	97.83	5.04	97.87	5.01	97.51	5.07	97.7	5.06	96.64	5.1	97.42	5.15	98.1	5.17	98.33
Q16	The lecturer was able to effectively take care of the students who attend class in-person and students who join the class virtually.	5.18	97.66	5.1	97.55	5.03	97.52	5.05	97.19	5.03	97.52	5.1	98.14	5.07	96.65	5.13	97.18	5.14	97.38	5.19	98.33
Q17	Overall speaking, I have very positive evaluation of the lecturer in this lecture.	5.12	97.86	5.06	97.44	5.02	97.63	5.07	97.75	5.05	97.4	5.1	98.04	5.09	97	5.11	97.42	5.17	97.74	5.2	97.94
Q18	The hybrid teaching of this lecture was very smooth.	4.96	95.94	4.98	96.78	4.96	97.11	5.03	97.42	5.01	96.85	5.1	97.93	5.07	96.64	5.09	97.28	5.14	97.27	5.18	97.95
Q19	The hybrid teaching enabled me to learn more efficiently about this lecture.	4.74	92.26	4.84	94.22	4.89	95.97	4.97	95.95	4.97	96.52	5.03	96.95	5.06	96.4	5.09	96.94	5.13	96.79	5.19	97.94
Q20	Compared to online teaching, the hybrid teaching approach provides me with more interaction opportunities with teachers and classmates.	4.76	91.48	4.83	92.8	4.9	94.73	4.97	95.17	4.97	95.72	5.03	96.41	5.03	95.7	5.07	96.71	5.12	96.56	5.15	96.66
Q21	Compared to the traditional teaching methods, the hybrid teaching approach provides me with more interaction opportunities with teachers and classmates.	4.53	84.36	4.67	87.86	4.75	90.2	4.88	92.36	4.88	93.35	4.95	93.98	4.95	92.71	5	93.79	5.08	95.01	5.11	95.12
Q22	Compared to the traditional teaching methods, the hybrid teaching approach increases my flexibility of access to the lecture.	4.8	91.87	4.89	94.12	4.9	94.94	5.02	96.18	5.01	96.85	5.06	96.5	5.08	95.82	5.12	96.25	5.17	96.66	5.19	97.42
Q23	Compared to traditional classroom learning, I learn better in hybrid learning-based environments.	4.44	83.08	4.65	87.38	4.73	90.67	4.87	92.23	4.89	93.27	4.97	94.53	4.97	92.68	5.03	94.96	5.08	95.01	5.12	96
Q24	Hybrid teaching allows me to learn the lecture materials according to my own situation.	4.8	94.76	4.91	96.35	4.94	96.59	5.04	97.75	5.01	97.64	5.09	97.92	5.07	96.3	5.12	97.07	5.15	97.5	5.21	97.94
Q25	I prefer hybrid learning to traditional learning.	4.17	73.46	4.44	80.37	4.62	87.06	4.77	89.34	4.78	90.44	4.89	92.68	4.92	91.64	5.01	93.31	5.06	93.81	5.07	93.94
Q26	I prefer hybrid learning to online learning	4.51	82.58	4.74	89.63	4.74	90.18	4.93	94.04	4.85	92.33	4.98	94.44	4.99	93.21	4.99	93.54	5.06	95.14	5.1	94.98
Q27	Overall speaking, I like the hybrid learning experience.	4.66	92.86	4.81	94.34	4.89	96.28	4.97	96.5	4.97	96.73	5.05	97.6	5.05	96.64	5.1	96.59	5.15	96.91	5.18	97.81
Q28	Overall speaking, I like the arrangement of this lecture (i.e., hybrid teaching and learning)	4.76	94.97	4.89	96.46	4.93	96.9	4.99	96.85	4.99	97.42	5.08	97.82	5.08	97.01	5.1	96.71	5.16	97.51	5.2	98.2

Note (Q1-Q28): All items were rated on a 6-point Likert scale with 1 = Strongly Disagree, 2 = Disagree, 3 = Slightly Disagree, 4 = Slightly Agree, 5 = Agree, 6 = Strongly Agree

For the perceptions of the lecture on law-abiding leadership (i.e., Lecture 8), findings similarly showed that the students were satisfied with the lecture (Table 3). First, more than 90% of the students were satisfied with the performance of the teachers, including their preparation, assistance to the students to understand the subject matter, as well as encouragement and care given to students. Second, the students were generally satisfied with the lecture design and lecture attributes, such as class atmosphere, student interaction, participation, and reflection opportunities. Third, students felt that the lecture helped them to understand the concept of law abidance and the HKNSL. Around 92% of the students agreed that they would strive to serve as socially responsible leaders. Fourth, students were satisfied with HyFlex teaching, including its efficiency and flexibility. Finally, the students perceived that the lecture was able to promote their personal development, including problem-solving, interpersonal communication, critical thinking, and understanding of the attributes of successful leaders, which are significant elements of well-being.

**Table 3:** Means and positive response rates of post-lecture evaluation for Lecture 8

Lecture		Lecture 8 (N=890)	
Questionnaire Item		Mean	Positive response rate%
Q1	The design of this lecture was very good.	4.69	85.46
Q2	The classroom atmosphere of this lecture was very pleasant.	4.70	85.47
Q3	This lecture increased my awareness of the importance of self-development.	4.61	83.45
Q4	This lecture has improved my problem-solving ability.	4.61	83.13
Q5	This lecture has improved my understanding of the importance of attributes of successful leaders (e.g., critical thinking, moral competence, etc.).	4.66	85.68
Q6	This lecture has improved my interpersonal communication skills.	4.66	84.67
Q7	There was much peer interaction amongst the students in this lecture.	4.74	86.95
Q8	This lecture has improved my critical thinking.	4.68	85.89
Q9	There was much student participation in this lecture.	4.79	88.59
Q10	There were many opportunities for reflection in this lecture.	4.72	87.25
Q11	This lecture is helpful to my personal development.	4.66	85.26
Q12	This lecture helps me understand the concepts of national security.	4.86	90.87
Q13	This lecture helps me understand the offenses and penalties surrounding the National Security Law in Hong Kong.	4.87	91.99
Q14	This lecture helps me understand the importance of law abidance in leadership.	4.73	89.6
Q15	Law abidance is important for the stability of a society.	4.87	90.7
Q16	I will try my best to serve as a socially responsible leader.	4.90	92.21
Q17	Overall speaking, I have very positive evaluation of this lecture.	4.71	86.64
Q18	The lecturer had a good mastery of the lecture material.	4.89	92.12
Q19	The lecturer used different methods to encourage students to learn.	4.89	92.09
Q20	The lecturer in this lecture was able to help students understand the knowledge covered in the lecture.	4.88	92.08
Q21	The lecturer was able to effectively take care of the students who attend class in-person and students who join the class virtually.	4.95	93.32
Q22	Overall speaking, I have very positive evaluation of the lecturer in this lecture.	4.90	92.44
Q23	The hybrid teaching of this lecture was very smooth.	4.94	93.42
Q24	The hybrid teaching enabled me to learn more efficiently about this lecture.	4.95	94
Q25	Hybrid teaching allows me to learn the lecture materials according to my own situation.	4.98	93.98
Q26	Overall speaking, I like the hybrid learning experience.	4.95	93.98
Q27	Overall speaking, I like the arrangement of this lecture.	4.84	89.94

Note (Q1-Q27): All items were rated on a 6-point Likert scale with 1 = Strongly Disagree, 2 = Disagree, 3 = Slightly Disagree, 4 = Slightly Agree, 5 = Agree, 6 = Strongly Agree

In addition to the quantitative responses, we also asked students to give comments on the lecture. Two general categories of responses emerged from the data. While the first category is related to “general comments” on the lecture characteristics (Table 4), the second category is specifically related to law-abiding leadership and the HKNSL (Table 5). Generally speaking, the comments are mostly positive with very few negative ones.

**Table 4:** Comments of the students on the lecture on law abidance leadership (N=85) with examples given under each category or sub-categories

Positive Responses (70 responses)	
*• “General Positive Comments” Category (13 responses)*: #Good. #Great. #Nice. #Very nice. #Such a great lecture! #I love it! #I think this is the best lecture I have had in this course.	
*• “Agreement with the Content, Support and Meaningfulness” Category (5 responses)*: #I totally agree with the content. #Support! #I think today’s lesson is educational and meaningful. #It’s really meaningful to learn in this lecture.	
*• “Enjoyment and Comfort” Category (6 responses)*: #I enjoyed the lecture a lot. #The lesson is interesting! #Enjoy this course sooooo much!! #Pleasant lecture.	
*• “Useful/Helpful/Gain Knowledge” Category (5 responses)*: #It is helpful. #Very useful knowledge. #It’s really useful to learn in this lecture. #Useful knowledge, many misconceptions are eliminated. #I now know more about the law.	
*• “Thanks, Appreciation and Regards to the Teacher and Assistant” Category (24 responses)*: #Thank you! #Thanks for using so much time to teach us today~( 3 h! you should be tired). #Thank you for such a pleasant lecture.	
*• “Good Classroom atmosphere / varied and interactive activities” Category (14 responses)*: #The atmosphere is very good, almost every student participated in the discussion. #Very interesting! #There are many interactions between teachers and students. #Very interesting atmosphere. #The atmosphere is not as scary as I thought. #The game in the lesson is fun and help us more engage in class. #There are many examples that are up to date.	
*• “Achievement” Category (3)*: #I can complete and pass the test after the lecture LoL Bravo! . #Very fruitful learning experience.	
**Neutral Responses (4): #**It’s good to know about what is really about in NSL. Though it’s sort of boring, it is our responsibility to know the law.	
**“Undecided” Responses (4): #**lot of. #integrity?	
**“Negative” Responses (7): #**The mic is too loud. #It is so cold here. #This lesson is too long for me. #Students may lose concentration on the lesson. #quite difficult. #I don’t like the topic.

**Table 5:** Comments of the students on the lecture on national security and the Hong Kong National Security Law (N=125) with examples given under each category or sub-categories

Positive Responses (124 responses)	
*• “Law abidance and law abidance Leadership” Category (40 responses)*: #I learned about why and how we obey the law. #Freedom does not means everything. We need to obey the law. #I have leant more about the importance of obeying the law. #We should follow the law since it is fundamental to our society. #Be a responsible resident. #The importance of being a law-abiding citizen and a functional member of society. #I reviewed law-abidance from a new perspective. #Through this lesson, I learnt more about the importance of obeying the law for ensuring the social stability in society. #Know that we should follow the law.	
*• “History and Politics Related to National Security” Category (5 responses)*: #About today’s class, I am very glad to review part of the history of China and learn about some politics status. #I now understand more about Hong Kong’s law and history. #I also learned a lot of knowledge of history of China and other country. #I got more about the history of HK.	
*• “Understanding and importance of National Security Category” (9 responses)*: #Have deep understanding of national security! Security is quite important. #The responsibility to protect the national security. #I think I know the importance of national security. #We should shoulder our responsibility. #Everyone takes a part to maintain the society a beautiful place.	
*• “Sense of Belonging to Hong Kong and China” Category (2 responses)*: #Able to learn in boarder sense of belonging to my country. Thank you. #In Hong Kong, I will always love this land. #It will always be peaceful, develop and belong to the five-star red flag.	
*• “Hong Kong National Security Law, Security Laws or NSL Category (68 responses)*: #Like the content of NLS, it’s important for us to know what we should not to do. #This lecture I learned many things and definition with some NSL examples, such as the four major crimes including secession, subversion, terrorism, as well as collusion with external forces. #I also knew that it is crucial for all of us to protect our country and city by NSL. #Enhances my understanding of NSL. #I feel safe under national security law. #I felt happy to hear different points of view from classmates on NSL. #I also reflected my views on NSL. #The prospect of Hong Kong is based on National security law. #NSL Goooooood!!!! #I like the content of National Security Law! # All Chinese should know NSL. #Today’s lesson gives us a more detailed explanation of NSL and let is to learn more about it. #Like to know NSL. #From this lecture, I have a deeper understanding of the four major crimes of NSL. #From this lecture, I know more about the national security law, also the difference between the reform and revolution. #I have learned different types of crimes and I didn’t know that a lot of countries have implemented NSL. Through this lecture, I have learned and gained deeper knowledge about laws. #I have a better insight about the NSL.	
**Negative Response (1 response)**: #Because of NSL, it is better not to say anything.	

Regarding predictors of overall evaluation of Lecture 8, three sets of multiple regression analyses were performed. In the first set where the “course” and “teacher” were the predictors and “overall evaluation” was the outcome, both quality of the “course” and “teacher” predicted “overall evaluation” (b = 0.44, p <.001 and b = 0.536, p < .001, respectively; F_(2/886)_ = 3518.47, p < .001; R^2^ = 0.89). In the second set of analysis, we found that both “law abidance” and “benefits” significantly predicted overall evaluation (b = 0.49, p < .001 and b = 0.47, p < .001, respectively; F_(2/886)_ = 427.78, p < .001; R^2^ = 0.85). Finally, we found that “course” (b = 0.17, p < .001), “teacher” (b = 0.44, p < .001), “law abidance” (b = 0.27, p < .001), and “benefits” (b = 0.13, p < .005) significantly predicted overall evaluation (F_(4/884)_ = 2,185.67; R^2^ = 0.91). These findings supported Hypotheses 3a to 3d.

## Discussion

In this study, we examined the perceptions of university students toward HyFlex teaching in a leadership subject in Hong Kong based on the post-lecture evaluation. Students generally had positive perceptions of the HyFlex mode of teaching. They perceived the lecture design, class atmosphere, instructors, and benefits in a favorable light. As the scientific literature on the evaluation of HyFlex mode of teaching is thin, our finding is a modest addition to the database.

As a new 3-hour lecture was designed and incorporated into the leadership subject as Lecture 8 in the 2021-22 academic year to promote law-abiding leadership and national security education among university students, we also specifically examined students’ views on this lecture regarding the overall lecture delivery and particular aspects related to national security education. The findings similarly revealed that the students had positive perceptions of the lecture design, class activities and interaction, lecture content, benefits, and overall evaluation of the lecture. Echoing the quantitative findings, qualitative findings also suggest that the lecture was well-received by students. The findings are interesting for three reasons. First, it demystifies the common belief that students would negatively experience national security law education. Second, the findings did not support the conjecture that the lecture would impair student well-being. On the contrary, the students agreed that the lecture promoted their psychosocial competence and law-abiding leadership knowledge, as well as behavioral intention to be socially responsible leaders. Third, there is no sign indicating that the students had the experience of being “brain-washed.” Instead, the students showed appreciation and felt that they had learned something meaningful in this lecture. Law abidance in college students is important because academic dishonesty such as plagiarism has intensified in the higher education sector (Jurdi et al., 2011; Park, 2003).

Nevertheless, we also need to be careful in interpreting the findings for several reasons. First, roughly one-quarter of the students did not respond to the post-lecture evaluation in Lecture 8, although we can argue that the current response rate of around 78% (890 in 1,146) is high enough for an online survey. Second, although the survey is anonymous, students may still hesitate to disclose their “real” feelings and they might simply give the “politically correct” answers. However, as the subject teachers had good relationships with the students, we did not find evidence for this possibility. Third, students’ satisfaction may just reflect their positive feelings of having an increase in knowledge and competence. We do not know whether the increase in knowledge and competence would lead to behavioral changes. Fourth, as the scope of law-abiding leadership and national security is very wide, we may have to fine-tune the content of the lecture. Furthermore, we have to answer the question of dosage — a sufficient amount of exposure is needed to create meaningful changes in students. Finally, we should take note of the debate on whether education in the school context is an effective way to promote law-abiding leadership.

For Lecture 8, we also investigated what aspects may contribute to students’ overall evaluation. Findings suggest that perceived subject design, teacher attributes, content on law-abiding leadership and national security, and benefits predicted the overall evaluation of the lecture on law-abiding leadership and national security. The present findings are generally consistent with the previous findings that perceived program design and teacher attributes predicted the program participants’ overall satisfaction with the program (Shek et al., 2017; Zhu & Shek, 2021). Additionally, the present findings showed that perceived benefits and quality of law-abiding leadership and national security education also predicted overall satisfaction. Hence, it is important to help students appreciate the benefits and value of the learning. One way is to encourage individual and group reflections amongst students on what they have learned and their assessment of their own changes after the lecture. Furthermore, as non-normative political participation such as non-law abidance is detrimental to one’s well-being (Ballard et al., 2020), it would be important to understand how law-abiding leadership contributes to the well-being of the students. As pointed out by Tonon (2020a), the inclusion of quality of life in social sciences is an important area to be considered.

The present study underscores the value of HyFlex teaching and particularly the use of HyFlex teaching to teach law-abiding leadership and national security within the framework of leadership. It also highlights the importance of conducting evaluation studies to understand the views of the students under HyFlex teaching. However, we have to note several limitations of the study. First, perceived benefits may not correspond to actual benefits in real life. Second, we only focused on one university in Hong Kong. As all universities have the responsibility to provide national security education and introduce HKNSL in Hong Kong, it would be important to understand the situation in other universities. Third, researchers should collect longitudinal data to understand how the knowledge and competence gained in the lecture may influence students’ law-abiding behavior over time. As the quality of life is an important issue to be considered in university teaching (Tonon, 2020a) and social sciences (Tonon, 2020b), it would be exciting to examine how law-abiding leadership and national security education may influence student well-being in the long run. Fourth, as the qualitative comments are brief, it would be fruitful to conduct focus groups and interviews with the students. Finally, in addition to students’ views, it would be equally important to understand teachers’ views in teaching law-abiding leadership and national security issues.
